# 

**Published:** 1924-01

**Authors:** 


### ORGANISING A HEALTH WEEK.

### A PRIZE OF £100.

"HEALTH Weeks" are a comparatively new departure. Here and there a great town has organised such a week, and there has recently been a very successful one in the Borough of St. Pancras, originated by the Acting Medical Officer of Health, Dr. Stella Churchill. Efforts of this kind are, however, still novel and distinctly occasional. To most people, indeed, the very name is unfamiliar. Yet all manner of "Weeks" and "Days" are constantly being organised for purposes which, however admirable, are mostly of small moment compared with the primary social subject of the health of the people. The State devotes a more minute attention than ever before to the physical well-being of the nation, but we are a sturdy and independent race, and we love to do things for ourselves and not to be beholden to Governments when by individual initiative we can help ourselves. The laws of health must needs be emphasised by the laws of the realm, but there is much that can be done outside Governments to make the sanitary code laid down by Nature better known and more carefully followed. We are only beginning to recognise the imperious character of this code, the observance of which would, in comparatively a few years, add enormously to the health, happiness and well-being of the people.

There is no better, no readier, no more popular means of impressing upon the public mind the duty, social and religious, of doing everything that in us lies—and every intelligent person can do much—not merely to reduce disease, but to prevent it. The medicine of the future will be mainly preventive medicine, and it is to prevention that "Health Weeks" primarily look. To teach the people how to preserve health, how to safeguard infant life, how to push death from disease farther and farther away is a noble object. And that, put into a nutshell, is the object of a "Health Week." Who, then, shall deny that there ought in every community in the country to be such a Week, always full of varied and fascinating interest, and always increasing in scope and attraction ?

It is the conviction of THE HOSPITAL AND HEALTH REVIEW that "Health Weeks" are destined to increase rapidly in number and to exercise a wide and powerful influence over the mind of the people. But they require to be carefully thought out, their details to be considered in the light of experience, and the incidents which compose them arranged with an eye to picturesque appeal. The newspaper, the pictorial poster the lecture, the film, the pageant, wireless, even music and singing can, and ought to be, pressed into the service. A Week of this kind should be a joyous Carnival, an event to be looked forward to with animated expectancy, and looked back upon with all the satisfaction of happy realisation. Instruction should be so imparted that a town or a district should long for the opportunity of acquiring knowledge of the laws of health and how to observe them. A dull or monotonous "Health Week" is certain to be a failure.

In the hope and in the confident expectation of popularising and greatly increasing the number of "Health Weeks" THE HOSPITAL AND HEALTH REVIEW is offering a prize of One Hundred Pounds for the best and most practical answer to the following three simple questions :—

1. How would you start a "Health Week" in your town or district ?
2. What would you make the principal features (naming not more than three) and why ?
3. How would you insure the success of your "Health Week" ?

We do not prescribe any length for these answers. They may be short or long, as the competitor pleases, but it is obvious that the most effective answers are likely to be crisp and compendious. A competitor may send in as many replies as he pleases, but each one must be accompanied by the coupon which will be found at the foot of page 34 of this issue. The competition will remain open until March 25, and we wish it to be clearly understood that it is open to all the world, men and women, doctors and laymen, health visitors and nurses. Everyone who has ideas on the subject is invited to give us the benefit of them. Each answer must bear the name and address of the competitor legibly inscribed. The best answer will be published, together with the name, address and portrait of the winner in our May number, which will be on sale on April 25. We reserve the right to publish any other answer which may be deemed to be sufficiently excellent. The decision of the Editor of THE HOSPITAL AND HEALTH REVIEW is to be accepted as final and conclusive.

### ANSWERS TO CORRESPONDENTS.

[In the course of every month we receive a number of enquiries upon subjects of interest to our readers. Hitherto these queries have been replied to by post, but since they often deal with matters of more than individual moment, we purpose in future to print the answers.]

Auto-Suggestion (Willesden). — The Coué Institute, 24, Grosvenor Place, S.W. 1.

W. H. J.—Apply for information to the College of Nursing, Henrietta Street and 7, Cavendish Square, W. 1.

Dispensing Bottles.—We think you would find Messrs. J. Isaacs and Co., of 106 Midland Road, N.W. 1, accurate in their measures and reasonable in their charges.

P. Ellis.—You are mistaken. The late Sir Frederick Treves was a Dorset, not a Devonshire man. He was born at Dorchester. We are sorry to have to administer this shock to your local patriotism.

Sister.—Yes ; potato peeling-machines are comparatively moderate in price. Messrs. Mabbett and Co., of 37-39 Old Street, E.C., and Poland Street, Manchester, for instance, manufacture one at £5 10s. which peels 6 lb. per minute.

F. J.—There is a very active Joint Hospitals Council at Sheffield, a model of its kind. The Secretary is Mr. S. R. Lamb, St. Peter's Close, Sheffield. There are similar organisations in other large towns, notably Birmingham, Manchester and Liverpool.

E. C.—Housekeeping courses can be obtained at Charing Cross Hospital; Brompton Hospital; the Hospital for Sick Children, Great Ormond Street, W.C. ; Swansea General and Eye Hospital ; Norfolk and Norwich Hospital, Norwich ; and Nottingham Hospital. Trained nurses can be given an administrative course at Guy's Hospital.

R. E. S.—As safety is your main object we would suggest your trying "Certo" Patent Firelighters. These are used in Government offices and are not dangerous to handle or store. They are made hygienically from sanitary materials, and can be obtained from Certo, Ltd., 142 High Street, Hampton Wick, Middlesex.

Lover of France.—We think you have confused the Chateâu de Chenonceaux, on the Cher, with the Chateâu d'Azay-le-Rideau on the Loire. The former now belongs to M. Menier, of chocolate fame ; the latter was sold some years ago by the Marquis de Biencourt for the bagatelle of £8,000 to the French Government, who intend to make it a museum of Renaissance furniture.

Contributory Schemes (To "L. S.," Chicago).—Many contributory schemes entitle the contributor to treatment as an out-patient. This is particularly the case in London, but though provincial schemes generally confine relief to in-patients, in many large towns where highly organised contributory schemes are in operation no out-patients' charges are made to contributors, save in maternity cases, when a woman is usually asked to give what she can afford. Additional information might be obtained from the note on Contributory Schemes published by the Voluntary Hospitals Commission (reference Volunteer Hospitals Commission 10), procurable at H.M. Stationery Office, Imperial House, Kingsway, W.C. 2.

F. H. S.—Admission as a Knight of Grace to the Order of St. John of Jerusalem in England is a reward for special service. Your name would have to be brought forward by a Knight of Justice of the Order, who would have been required to produce a full record of your services to the Order or its departments, and to certify that you profess the Christian faith. Knights of Grace are eligible for appointment by the Grand Prior to any executive office or assistant executive office in the Order, or to serve on the Chapter-General, Council, or any committee. The badge of a Knight of Grace is a Maltese Cross, of white enamel, set and embellished in silver, suspended from a black watered riband round the neck. A Knight of Grace may wear on the left side, below the breast, a star, consisting of the Maltese Cross of white enamel, set and embellished in silver. The mantles of the Knights of Grace, if members of Chapter, are of black merino, faced with black silk. On the left side is the Maltese Cross, twelve inches in diameter, in white linen, the embellishment of the lions and unicorns being embroidered in white silk. The cordons and tassels are of black mohair. The grades are Knights and Ladies of Justice, Knights and Ladies of Grace, Chaplains and Esquires. The work of the Order consists of the control of the St. John Ambulance and Brigade and of the British Ophthalmic Hospital in Jerusalem.

### RECENT APPOINTMENTS.

### Medical Officers, Chaplains, &c.

Barron, D. C., M.D., Ch.B., B.Sc. ; Assistant Lecturer in Medicine, University of Sheffield.

Clark, W. R. Le Gros, L.R.C.P., F.R.C.S., Reader in Anatomy, St. Bartholomew's Hospital School.

Coleman, C. J., M.A., M.D., D.P.H., M.O.H., Lincoln ; M.O.H. and School M.O., Holland County Council, Lincs. £1,000 and travelling expenses.

Dalby, Marjorie E., M.B.B.S., D.P.H., Temporary Assistant M.O., Willesden.

Dent, Patricia, M.D., B.S., Maternity and Child Welfare Officer, Leicester.

Morrell, R. A., M.R.C.S., L.R.C.P. ; Lecturer in Radiology University of Sheffield.

Skerritt, J. H., L.D.S. ; Lecturer on Operative Dental Surgery, University of Sheffield.

Turner, Mary, M.B., Ch.B., Assistant M.O.H., Chester.

Wilkinson, G., M.B., B.C., F.R.C.S. ; Lecturer in the History of Medicine, University of Sheffield.

Wilson, Horace, M.R.C.S., L.R.C.P., D.P.H., Tuberculosis Officer and Acting M.O., Southwark ; M.O.H., Southwark. £1,000.

### Public Health and Hospital Appointments.

Brebner, M. G., Matron, Inverness-shire Sanatorium, Fort Augustus.

Cameron, Anabell, Assistant Matron British General Hospital; Matron, Herefordshire General Hospital.

Fenton James, M.B., Ch.B. M.O.H., Kensington ; Examiner, Sanitary Inspectors' Examination Board, 1924.

Fletcher, G. B. E., Town Clerk, Islington ; Examiner, Sanitary Inspectors' Examination Board, 1924.

Martin, Arthur J., Examiner, Sanitary Inspectors' Examination Board, 1924.

Russell, Helen I., Queen's Nurse, Guildford; School Nurse, Staffordshire Education Committee.

Shenton, Nellie, Dunster Cottage Hospital ; School Nurse, Staffordshire Education Committee.

Shipperbottom, Annie, Manchester Health Department, Health Visitor and School Nurse, Lowestoft.

Wankwill, A. T., M.R.C.S., M.D., M.O.H., Hornsey ; Examiner, Sanitary Inspectors' Examination Board, 1924.

White, Mary Gregory ; Superintendent School Nurse, Walthamstow.

Williamson, Edith Ethel ; School Nurse, West Ham.

### A Hot Water Bottle.

A hot water bottle is in these cold days more of a necessity than a luxury, and a rubber one that can be hugged is certainly the most comforting kind. The worst of this type is that its life is often rather brief, but Ingram's "Eclipse" Hot Water Bottle may be depended upon to give good service for some years. Indeed, "G. M. W." writes : "I have used it constantly for four years, and it has never given me the slightest trouble ; its big, flat, round stopper has never leaked or wanted a new washer." This patent washer cannot be lost and the patent neck is guaranteed not to leak. The bottle can be obtained from all good chemists and can be thoroughly recommended.

"THE HOSPITAL AND HEALTH REVIEW"

### Managerial Notices.

*All letters on Business matters must be addressed to the Manager, "The Hospital and Health Review," 28 and 29 Southampton Street, London, W.C. 2.*

RATES FOR SUBSCRIPTION (payable in advance).

| | | | |
|---|---|---|---|
| Three Months (including Postage) | .. .. | 1s. | 9d. |
| Six Months do. | .. .. | 3s. | 6d. |
| Twelve Months do. | .. .. | 7s. | 0d. |

**It is especially requested that remittances be made by Cheque or Postal Order and not Stamps.**

Subscriptions, which may commence at any time, are payable in advance. Cheques and Post-Office Orders should be crossed Westminster Bank, Covent Garden Branch, and made payable to the Manager of THE HOSPITAL AND HEALTH REVIEW.

***Advertisements.*** Scale of charges and particulars of special positions may be obtained on application to the Manager.

*SUBSCRIPTION FORM.*

*Please send me* THE HOSPITAL AND HEALTH REVIEW *for.............. months, commencing with your issue of ................, for which please find enclosed ........*

*Name* ....................................

*Address* ....................................

